# Left Ventricular Diastolic Function in Children with Atrial Septal Defects Improves After Closure by Means of Increased Hydraulic Force

**DOI:** 10.1007/s00246-024-03534-5

**Published:** 2024-06-11

**Authors:** Pia Sjöberg, Henning Clausen, Håkan Arheden, Katarina Steding-Ehrenborg, Petru Liuba, Erik Hedström

**Affiliations:** 1 Clinical Physiology, Department of Clinical Sciences Lund, Lund University, Lund, Sweden; 2 Department of Clinical Physiology, Skåne University Hospital, 22185 Lund, Sweden; 3 Department of Paediatric Cardiology, Children’s Heart Centre, Skåne University Hospital, Lund, Sweden; 4 Paediatrics, Department of Clinical Sciences Lund, Lund University, Lund, Sweden; 5 Diagnostic Radiology, Department of Clinical Sciences Lund, Lund University, Lund, Sweden; 6 Department of Radiology, Skåne University Hospital, Lund, Sweden

**Keywords:** Congenital heart defect (CHD), Cardiac magnetic resonance imaging (CMR), Physiology, ASD closure

## Abstract

Hydraulic force aids diastolic filling of the left ventricle (LV) by facilitating basal movement of the atrioventricular plane. The short-axis atrioventricular area difference (AVAD) determines direction and magnitude of this force. Patients with atrial septal defect (ASD) have reduced LV filling due to the left-to-right shunt across the atrial septum and thus potentially altered hydraulic force. The aims were therefore to use cardiac magnetic resonance images to assess whether AVAD and thus the hydraulic force differ in children with ASD compared to healthy children, and if it improves after ASD closure. Twenty-two children with ASD underwent cardiac magnetic resonance before ASD closure. Of these 22 children, 17 of them repeated their examination also after ASD closure. Twelve controls were included. Left atrial and ventricular areas were delineated in short-axis images, and AVAD was defined as the largest ventricular area minus the largest atrial area at each time frame and normalized to body height (AVADi). At end diastole AVADi was positive in all participants, suggesting a force acting towards the atrium assisting the diastolic movement of the atrioventricular plane; however, lower in children both before (6.3 cm^2^/m [5.2–8.0]; *p* < 0.0001) and after ASD closure (8.7 cm^2^/m [6.6–8.5]; *p* = 0.0003) compared to controls (12.2 cm^2^/m [11.3–13.9]). Left ventricular diastolic function improves after ASD closure in children by means of improved hydraulic force assessed by AVAD. Although AVADi improved after ASD closure, it was still lower than in controls, indicating diastolic abnormality even after ASD closure. In patients where AVADi is low, ASD closure may help avoid diastolic function deterioration and improve outcome. This could likely be important also in patients with small shunt volumes, especially if they are younger, who currently do not undergo ASD closure. Changes in clinical routine may be considered pending larger outcome studies.

## Background

Patients with an atrial septal defect (ASD) have a left-to-right shunt across the atrial septum, which leads to right ventricular volume overload and decreased left ventricular filling. The reduced left ventricular filling leads to a relatively smaller ventricle in patients with ASD compared to healthy individuals [1]. Further, left ventricular function is affected up to 18 years after ASD closure [2–4]. However, the importance of reduced ventricular filling and mechanisms behind decreased function over time in patients with ASD is insufficiently understood. More knowledge is needed to be able to optimize treatment, particularly in children.

**Fig. 1:**
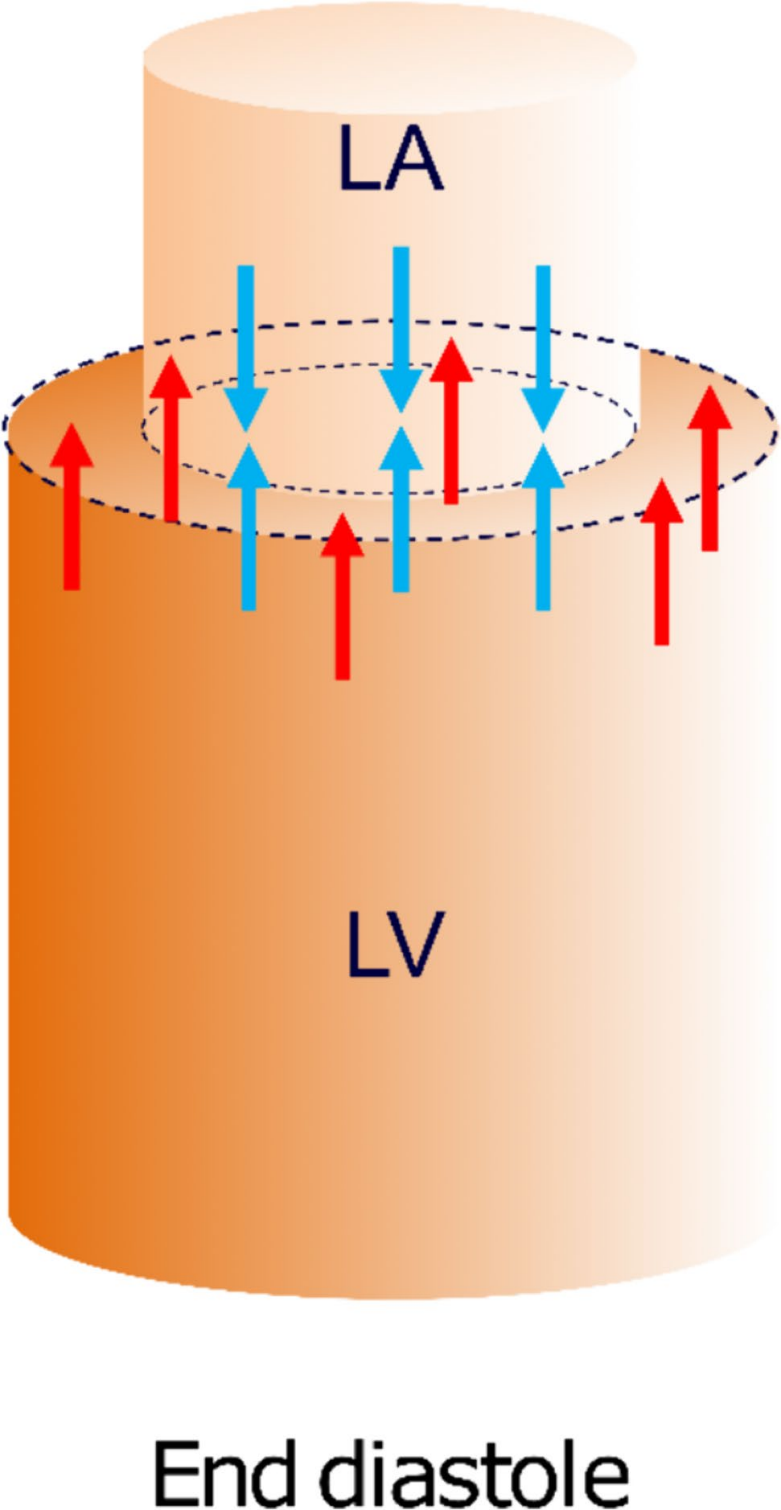
Schematic illustration of the left side of the heart. The cylinders represent the left side of the heart at end diastole with a large left ventricle (LV) at the bottom and a small left atrium (LA) at the top. The arrows represent the forces the blood exerts on the atrioventricular plane in the atrium and in the ventricle. The blue arrows counterbalance each other, whereas the red arrows represent the net force which pushes the atrioventricular plane upwards. The net force depends on the difference in area of the atrioventricular plane. A larger difference will result in a higher net force. The magnitude of the hydraulic force is thus proportional to the atrioventricular area difference or AVAD which is defined as ventricular short-axis area—atrial short-axis area

Diastolic filling of the left ventricle is affected by the geometrical relationship between the left ventricle and atrium [5, 6] (Fig. 1). The blood pressure acts on the walls of the atrium and the ventricle. According to Pascal’s law the force on the walls equals the pressure multiplied by the area and when the mitral valve is closed the force against the walls is the same in all directions. During diastole, when the mitral valve is open, the pressures in the left ventricle and atrium are almost equal [6]. The movement of the base and the apex of the heart is restricted, but the AV-plane has the possibility to move. Thus, when the ventricular short-axis area is larger than the atrial short-axis area, the net force will be acting toward the atrium and, when the mitral valve is open, thus supporting motion of the atrioventricular plane toward the atrium. Since force is the product of pressure and area, the hydraulic force acting on the atrioventricular plane will be proportional to the short-axis atrioventricular area difference (AVAD). This hydraulic force adds to the forces of elastic recoil, generated by ventricular contraction, creating a pressure difference between the atrium and ventricle [6]. A previous study on adult patients after heart transplantation showed impaired hydraulic force due to larger left atria [7]. As patients with ASD have a smaller left ventricle than normal [8–10], but not necessarily a differently sized atrium, this may impact the hydraulic force and thus diastolic function.

The aims of this study were therefore to assess (1) if the hydraulic force differs in children with ASD compared to healthy volunteers, (2) whether it contributes to left ventricular diastolic dysfunction in presence of ASD, and (3) whether hydraulic force changes in direction or magnitude after ASD closure.

## Method

The study was approved by the Swedish Ethical Review Authority (2019-05490) and follows the principles of the Declaration of Helsinki. Written informed consent was obtained from the participants’ parents, with the child’s wish to participate considered. The study is reported according to the STROBE guidelines [11].

Children referred to our tertiary center for congenital heart defects for elective treatment of ASD or ASD with partially anomalous venous drainage under the age of 18 years were eligible for the study. Prospective recruitment was through non-random methods based on chronological order of planned treatment. Children unable to comply with cardiac magnetic resonance (CMR) without general anesthesia or with contraindications to CMR were excluded. Twenty-three children with ASD were included from March 2021 to February 2023. The number of patients was based on sample size calculation by Bellenger et al., taking into account the high precision of CMR [12]. Clinical data were reviewed, including standard transthoracic echocardiography prior and after treatment. They underwent CMR before intervention and between 6 and 12 months after closure when ventricular remodeling was assumed to be normalized based on previous studies [3, 13, 14]. Six children with ASD received sedation before CMR as per local clinical practice guidelines with dexmedetomidine at 2–3 µg/kg/dose intranasally (Dexdor^©^, Orion Pharma, Espoo, Finland). Twelve healthy volunteers were included as controls.

### Cardiac Magnetic Resonance Imaging

Examinations were performed using a 1.5 T scanner (Aera, Siemens Healthineers, Erlangen, Germany). Clinical standard balanced steady-state free precession short-axis cine images covering the whole heart were acquired with retrospective ECG gating. Two-dimensional free-breathing through-plane phase-contrast flow measurements were performed in the ascending aorta in all participants to quantify the effective stroke volume. Also, pulmonary artery flow was measured in patients to quantify the left-to-right shunt.

### Image Analysis

All image analyses were performed using Segment software (http://segment.heiberg.se) [15]. Left ventricular and atrial endocardial borders, including the atrial appendage, were delineated in all timeframes for left ventricular and atrial volumes and for calculation of time-resolved AVAD. Right ventricular endocardial borders were delineated in end systole and end diastole for volumes. Cardiac volumes were indexed to body surface area to be comparable between studies. Analysis of AVAD has been previously described in detail [5]. In short, AVAD was defined as the difference between the largest ventricular short-axis area minus the largest atrial short-axis area at each time point during the cardiac cycle. To be able to compare AVAD between patients and controls, AVAD was assessed at end systole, and at the end of diastole. Since the hydraulic force only can support the motion of the atrioventricular plane during diastole when the mitral valve is open and pressures in atrium and ventricle similar, for didactic reasons AVAD at end systole is throughout this work denoted “the beginning of diastole”. AVAD was normalized to height (AVADi) to be able to compare participants of different sizes. Height is preferred over body surface area to avoid bias in case of a large variation in body mass index between participants [16]. The area under the curve (AUC) for AVADi during diastole was calculated as a measure of how much of the hydraulic force is working in favor of moving the atrioventricular plane toward the base of the heart, i.e., aiding diastolic filling of the left ventricle. Also, the proportion of the duration of diastolic AVADi ≥ 0 was measured as a percentage of diastolic duration.

### Statistical Analysis

Statistical analyses were performed using Graph Pad Prism 10 (GraphPad Software, Boston, Massachusetts, USA). Continuous variables are presented as median [interquartile range] according to lack of normal distribution as determined by histograms. Mann–Whitney’s rank test compared AVAD between children with ASD and controls, and Wilcoxon’s signed rank test compared AVAD in children before and after ASD closure. *P* < 0.05 was considered to show significant differences.

## Results

**Fig. 2:**
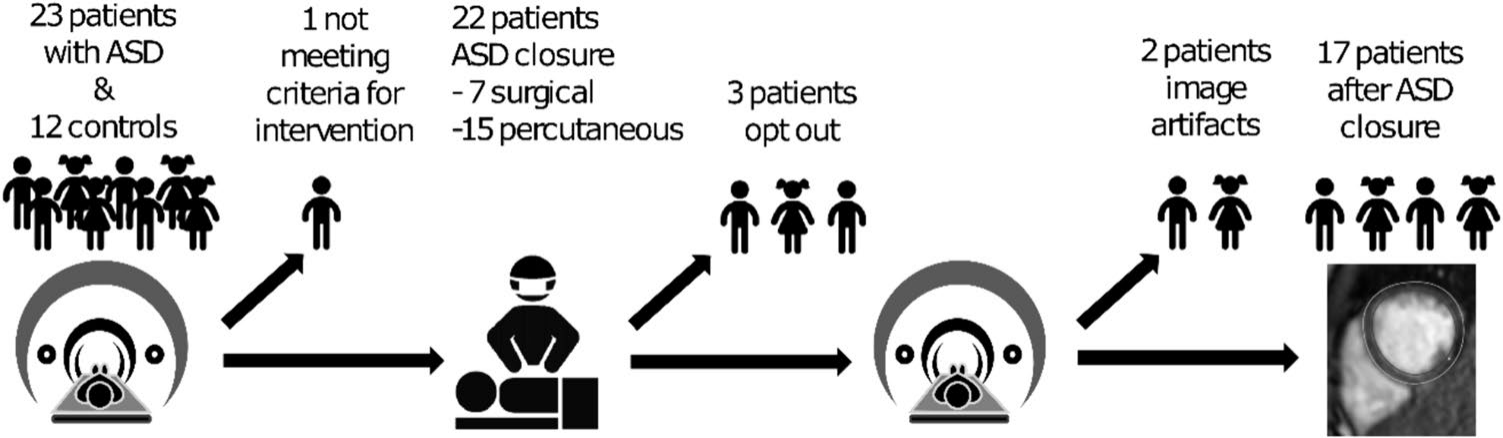
Flow chart of inclusion. Twenty-three children with ASD and 12 healthy controls were examined with cardiac magnetic resonance imaging (CMR). One was excluded due to not meeting criteria for intervention due to a shunt < 1.5/1. ASD was closed in 22, 7 surgically and 15 percutaneously. Three patients did not want to participate in the postoperative examination. The short-axis cine images in two postoperative examinations were judged by two senior CMR physicians to have too many artifacts due to patient motion and the patients were excluded from follow-up analyses leaving 17 patients to be analyzed after ASD closure. (Parts of figure: Clin Physiol Funct Imaging. 2022; 1–8)

Figure 2 shows the patient inclusion flow chart. Twenty-three children with ASD were examined before ASD closure. One child did not meet the criteria for ASD closure and was thus excluded from the group analysis. Seventeen children were examined and analyzed after ASD closure. One child included in the study had partial anomalous venous drainage from the right lung and another child anomalous venous drainage from the left lung which were corrected at the time of surgery. None of these cases had signs of pulmonary venous obstruction or pulmonary hypertension nor obstructive left-sided lesions, such as mitral stenosis, based on echocardiographic findings. Twelve healthy children acted as controls.

Table 1 shows patient characteristics, type of ASD, type of ASD closure and cardiac parameters. Body mass index in the patient group varied from 13.4 to 33.6 kg/m^2^ and in the control group from 13.9 to 19.5 kg/m^2^.

**Table 1:** Patient characteristics and cardiac parameters

	Children with ASD before closure (*n* = 22)	Children after ASD closure (*n* = 17)	Controls (*n* = 12)
Time from 1st CMR to ASD closure (days)	1 [1–1]	–	–
Time from ASD closure to 2nd CMR (months)	–	7 [6–9]	–
*Q*_p_/*Q*_s_	1.8 [1.6–2.4]	–	–
ASD secundum	21 (95%)	–	–
Sinus venosus defect	1 (5%)	–	–
Surgical closure	7 (32%)	–	–
Percutaneous closure	15 (68%)	–	–
Age (years)	8 [5–13]	11 [7–14]	9.5 [7–12]
Sex (%men)	7 (32%)	6 (35%)	7 (58%)
BSA (m^2^)	1.1 [0.8–1.5]^####^	1.3 [1–1.7]	1.1 [0.9–4]
Height (cm)	137 [110–160]	144 [128–165]	140 [128–163]
Heart rate (bpm)	83 [75–94]**	77 [64–99]	75 [68–79]
Left ventricle			
EDVi (ml/m^2^)	63 [57–70]**** ^###^	76 [70–86]	82 [78–89]
ESVi (ml/m^2^)	26 [22–30]**** ^##^	30 [27–33]	35 [31–38]
Ejection fraction (%)	60 [54–64]	60 [54–65]	58 [54–63]
Cardiac index (l/min/kg)	3.3 [2.7–3.6]^#^	3.5 [2.9–4.2]	3.6 [3.2–4.0]
Left atrium			
EDVi (ml/m^2^)	14 [11–17]	14 [12–19]	13 [11–15]
ESVi (ml/m^2^)	34 [26–37]	35 [24–42]	34 [26–36]
Right ventricle			
EDVi (ml/m^2^)	130 [111–160]**** ^####^	80 [82–101]	87 [81–93]
ESVi (ml/m^2^)	55 [48–66]**** ^###^	41 [34–51]	36 [35–41]
Ejection fraction (%)	58 [54–62]^##^	53 [46–60]	57 [52–61]

Numbers are presented as or median [IQR]

*ASD* atrial septal defect, *Q*_p_ pulmonary blood flow, *Q*_s_ aortic blood flow, *EDVi* end-diastolic volume indexed to body surface area, *ESVi* end-systolic volume indexed to body surface area

Patients with ASD vs controls: *p < 0.05, **p < 0.01, ****p < 0.0001; Patients before vs after ASD closure: ^#^*p* < 0.05, ^##^*p* < 0.01, ^###^*p* < 0.001, ^####^*p* < 0.0001

**Fig. 3:**
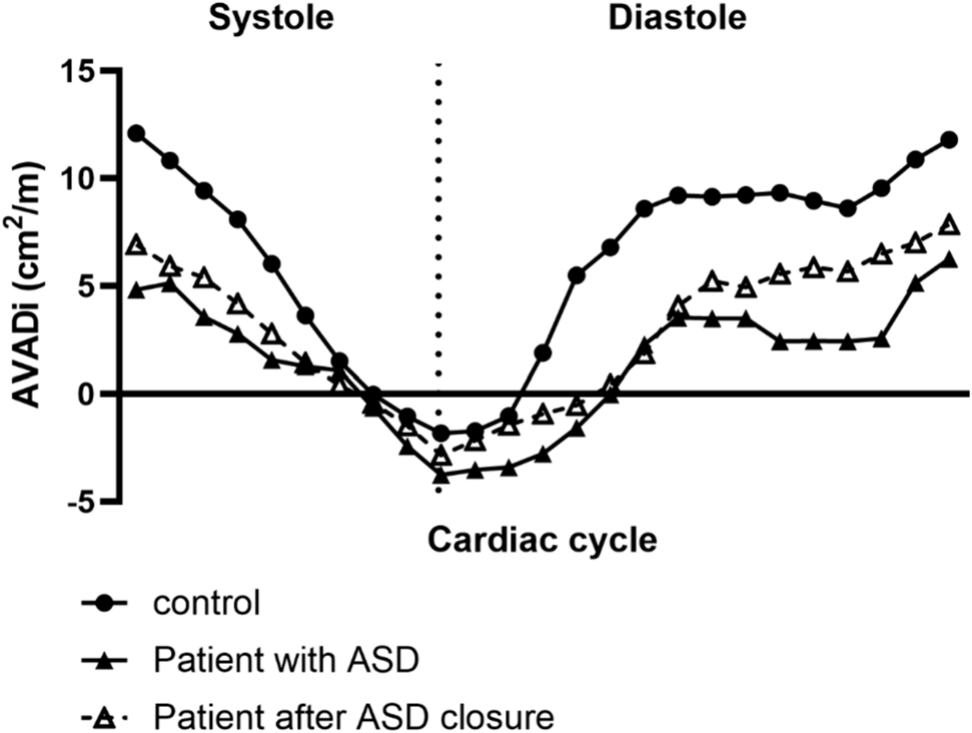
Examples of atrioventricular area difference (AVAD) during the cardiac cycle in a child with ASD before and after ASD closure and in a control. The graph visualizes that AVAD is negative at the beginning of diastole, thus there is a hydraulic force that acts towards the ventricle for a short period before AVAD increases and are positive during most of diastole. A positive AVAD will result in a hydraulic force pushing the atrioventricular plane the atrium, thus facilitating left ventricular filling. Peak AVAD and the area under the curve during diastole is, however, lower in patients suggesting a lower hydraulic force in patients

**Fig. 4:**
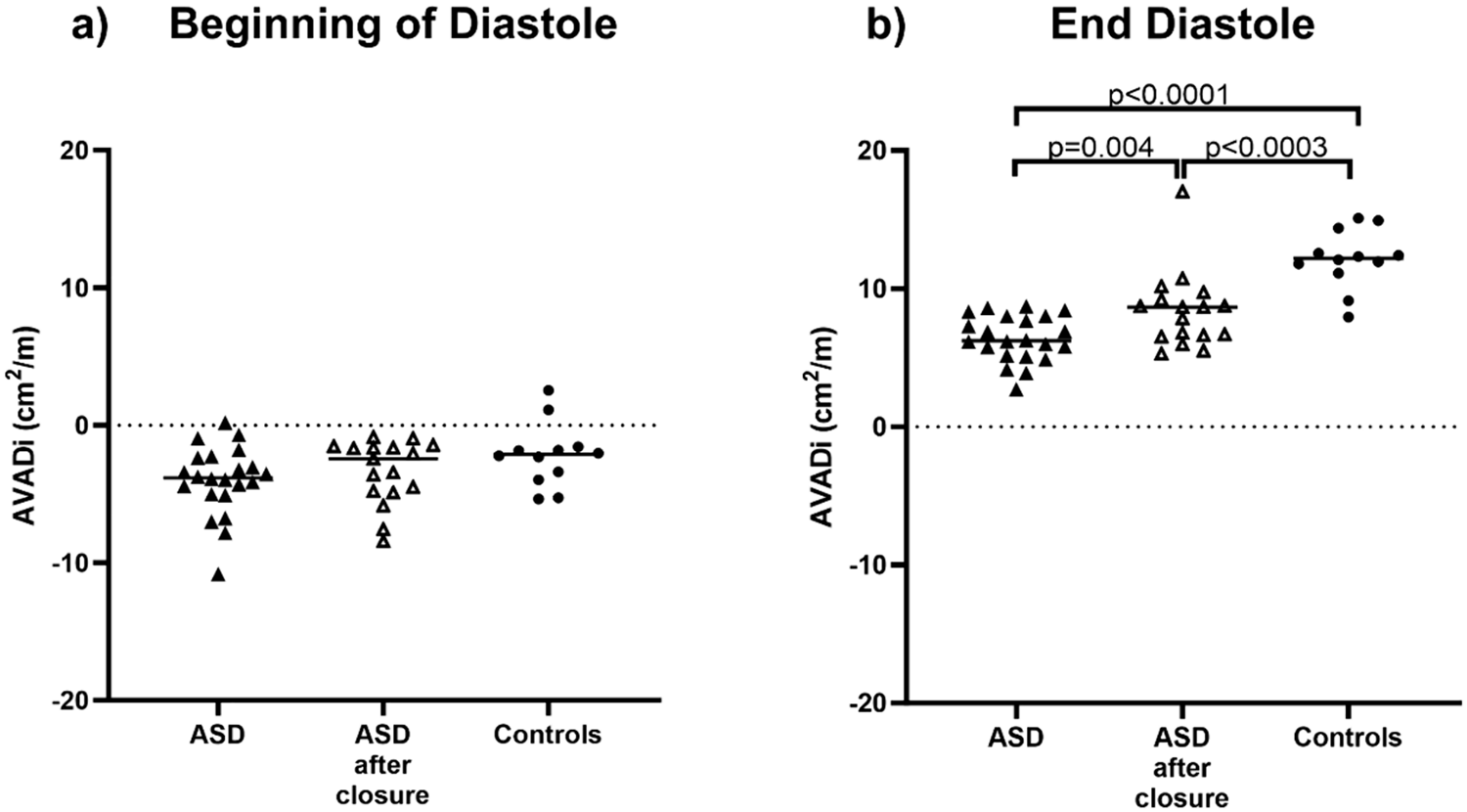
Atrioventricular area difference indexed to height (AVADi) in the left ventricle at the beginning of diastole and at end diastole in children with atrial septal defect (ASD) before and after ASD closure and in controls. **a** At the beginning of diastole AVADi is negative in all participants except for two controls and one child with ASD and thus there is a hydraulic force acting towards the apex. **b** At end diastole AVADi is positive in all participants and thus the hydraulic force is directed towards the base of the heart. This means that it facilitates atrioventricular plane movement and filling of the left ventricle. Children with ASD had lower AVADi than controls and although AVADi increased after ASD closure, AVADi did not increase to the values shown for controls

Figure 3 shows examples representative for the median values of time-resolved AVADi throughout the cardiac cycle in a patient before and after ASD closure and in a control. Figure 4 shows indexed AVAD at the beginning of diastole and at end diastole. At the beginning of diastole there was no difference in AVADi between children with ASD (−3.8 [−2.4 to −5.0] cm^2^/m) compared to controls (−2.1 [−1.6 to −3.8] cm^2^/m); *p* < 0.07) or between children after ASD closure (−2.4 [−1.6 to −4.8] cm^2^/m) and controls (−2.1 [−1.6 to −3.8] cm^2^/m; *p* = 0.59), Table 2. At end diastole, AVADi was positive in all participants, however, lower in children with ASD (6.3 [5.2–8.0] cm^2^/m) than in controls (12.2 [11.3 to 13.9 cm^2^/m], *p* < 0.0001). Further, although AVADi increased in children after ASD closure (6.2 [5.1 to 8.0] cm^2^/m vs 8.7 [6.6 to 9.5] cm^2^/m; *p* = 0.004), it did not normalize compared to controls (8.7 [6.6 to 9.5] cm^2^/m vs 12.2 [11.3 to 13.9] cm^2^/m; *p* < 0.0003).

**Table 2:** Short-axis left atrioventricular area difference (AVAD) indexed to body height

	Children with ASD (*n* = 22)	Children after ASD closure (*n* = 17)	Controls (*n* = 12)
AVADi (cm^2^/m) at the beginning of diastole	− 3.8 [− 2.4 to − 5.0]	− 2.4 [− 1.6 to − 4.8]	− 2.1 [− 1.6 to − 3.8]
AVADi (cm^2^/m) at end diastole	6.3 [5.2–8.0]****	8.7 [6.6–9.5]**** ^*##*^	12.2 [11.3–13.9]

Numbers are presented as median [IQR]

*ASD* atrial septal defect, *AVADi* atrioventricular area difference indexed to body surface area

****p < 0.0001 ASD before or after closure vs controls; Patients before vs after ASD closure: ^##^*p* < 0.01

The proportion of the duration of diastolic AVADi ≥ 0 was lower in children with ASD (76 [62–84]%) compared to controls (87 [80–88]%; *p* = 0.0004). After ASD closure this proportion increased from 76 to 80% [71–86]% (*p* = 0.011) but did not reach values in controls (87 [80–88]%; *p* = 0.056).

The AVADi AUC during diastole was lower in children with ASD (0.29 cm^2^/m × ms [0.16–0.59]) compared to controls (1.0 cm^2^/m × ms [0.83–1.34]; *p* < 0.0001). After ASD closure AUC during diastole increased from 0.29 to 0.48 cm^2^/m × ms [0.37–0.92] (*p* = 0.002) but did not reach values in controls (1.0 cm^2^/m × ms [0.83–1.34]; *p* = 0.0038).

## Discussion

This study shows that left ventricular diastolic function improves towards normal values after ASD closure in children by increasing the hydraulic force, as evaluated by AVADi. Although AVADi improved after ASD closure, it was still lower than in controls 7 months thereafter. The decreased left ventricular filling may contribute to decreased exercise capacity [8, 17]. It may be hypothesized that earlier ASD closure, improving diastolic filling, improves long-term outcome. Therefore, in patients where atrioventricular area difference is low, ASD closure may help avoid diastolic function deterioration and improve outcome. This is likely important also in patients with small shunt volumes, who currently do not undergo ASD closure. Changes in clinical routine may be considered pending larger outcome studies.

The current study shows that both children with ASD and controls have negative AVADi in the beginning of diastole suggesting a hydraulic force acting towards the ventricle. Early in diastole, AVADi becomes positive leading to a change of direction of the hydraulic force, which during the main part of diastole thus works towards the atrium. This means that the movement of the atrioventricular plane towards the base of the heart is facilitated and filling of the left ventricle during diastole is augmented. Although this mechanism is valid both in children with ASD and in controls, the current study also shows that AVADi at end diastole is lower in patients after ASD closure as compared to controls. In children with ASD, the time with positive AVADi and thus a hydraulic force towards the base of the heart augmenting diastolic filling, was shorter during diastole than in controls. Together with a higher heart rate in children with ASD, these mechanisms suggest that diastolic dysfunction in children with ASD is likely augmented by a reduced hydraulic force.

The anatomical reason for a decreased AVADi in patients with ASD is mainly the small left ventricle, since the size of the atrium is normal, as confirmed in the current study. This is in line with previous studies showing that patients with ASD have diastolic impairment and increased sympathetic tone, which is likely related to the underfilled left ventricle [4, 8, 18]. The atrial function also affects the AVAD, reflected by an increase in AVAD at late diastole caused by the atrial contraction, as shown in the examples in Fig. 3.

After ASD closure, the left ventricle increased in both end-systolic and end-diastolic volumes as preload increased, in line with previous studies [19]. Low left ventricular compliance and abnormal filling properties have been reported several years after closure in this context [20], which might also explain the decreased exercise capacity these patients experience decades after ASD closure [2, 8]. In patients with ASD left ventricular volume decreases with age with a concomitant decrease in stroke volume and diastolic function but disproportionate to left ventricular mass [21]. These age-related changes, in addition to left ventricular remodeling due to LV underfilling related to the left-to-right shunt in patients with ASD, predispose the low AVADi before ASD closure. In the current study AVADi was still lower in patients after ASD closure than in controls despite that the patients had normal atrial and ventricular volumes after ASD closure. This suggests that the shape of the left ventricle and/or atrium might also be abnormal as AVAD is based on the area differences and not volumes.

In the current study of children and adolescents, the ASD lesion was mainly an isolated defect, and only in rare cases associated with partial anomalous pulmonary venous drainage without obstruction. There was no sign of clinically apparent arterial or pulmonary hypertension based on clinical assessments, including echocardiography. Further, there was no additional right-sided heart lesion that otherwise could have altered right atrial pressure, nor any left-sided obstructive lesions such as mitral stenosis. These relatively young patients had no left atrial enlargement, which otherwise seems do develop over time [19, 22]. A dilated left atrium in addition to a small left ventricle would diminish the hydraulic force even more and might thus further increase diastolic dysfunction over time. A small restrictive left ventricle could also explain the increased atrial pressure seen at exercise, and often already at rest [20], which predisposes to the increased risk for atrial enlargement and thus atrial fibrillation with increasing age.

The independent prognostic value of AVADi in pediatric patients with ASD and after ASD closure remains to be investigated. Nevertheless, a decrease in AVAD and thus a decrease in hydraulic force, assessed by echocardiography in adults with preserved systolic left ventricular function but with diastolic dysfunction, was linked to increased overall mortality at 5-years follow-up with a hazard ratio of 20–33% [23]. Decreased hydraulic force was also shown to have adverse prognostic value beyond the common measures of diastolic dysfunction in that population of more than 5,000 patients [23]. Thus, it may be hypothesized that AVAD will add prognostic information also in patients with ASD.

According to the current guidelines for ASD closure, the indication relies on the echo-assessed size of the right atrium and right ventricle based on echocardiography. Including AVAD data for decision making could be of particular importance in patients with smaller ASD’s and borderline right atrial and right ventricular sizes. The current study suggests that ASD closure may be beneficial when AVADi is low even if the left-to-right shunt does not cause right ventricular dilatation and, according to the current guidelines, is considered hemodynamically non-significant [24]. One child in this study had a shunt volume below treatment threshold and was therefore excluded from the case group analysis. A separate analysis, however, showed lower AVADi (8.5 cm^2^/m) than in controls, which suggests that the left ventricle actually was affected in this child. Since the hydraulic force contributes to diastolic filling, ASD closure leading to an increase in AVAD as shown in the current study may be assumed to substantially improve diastolic function. This also agrees with the findings of Maagaard et al. showing reduced cardiac index during exercise in adults with unrepaired ASD and small or spontaneously closed ASD [25]. As of today there are no pediatric reference values for AVADi but the current study suggests, although the population is limited in numbers and spread of age, that the lower reference value is approximately 10 cm^2^/m, which means that it is likely similar to that of adults [5]. The current results also support ASD closure at a young age to minimize the duration of an underfilled left ventricle. Future longitudinal studies might reveal if AVAD has additional prognostic information in patients with ASD.

## Limitations

Comparison between non-invasive and invasive measures of hydraulic force were not performed, as invasive assessment of left-sided hemodynamics was not routinely performed during cardiac catheterization or open-heart surgery since that would have prolonged procedure time and increased radiation exposure. Invasive cardiac catheterization in healthy children or at follow-up after ASD closure was not considered to be ethical. Further, the invasive measurement would have been performed under different hemodynamic circumstances e.g., under general anesthesia in fasting conditions, which could have influenced diastolic filling pressures. There were more girls in the patient group. How sex affects AVADi is not known and has to be addressed in a larger population of healthy volunteers. Also, the median follow-up time after ASD closure is 7 months in this study. Later remodeling of the heart may take place that could affect the AVADi, which needs to be assessed in a separate follow-up study. Two patients had partially anomalous venous return, which could possibly affect the results. However, these patients had AVAD values similar to the rest of the population. Cardiopulmonary exercise testing was not part of the study protocol, why analysis of possible exercise improvement after ASD closure was not performed.

## Conclusion

Left ventricular diastolic function improves after ASD closure by means of an increased hydraulic force as evaluated by atrioventricular area difference. Although this force improved after ASD closure, it was still lower than in controls 7 months thereafter. This study shows proof-of-concept how the diastolic mechanism AVAD may affect filling in this patient group. If atrioventricular area difference is low, ASD closure may avoid diastolic function deterioration and improve outcome. This is likely important also in patients with small shunt volumes, who currently do not undergo ASD closure. Changes in clinical routine may be considered pending larger outcome studies.
